# Distribution of Major HLA-A, -B, -DR, and -DQ Loci Potentially Associated with Multiple Sclerosis in a Healthy Population from Southern Morocco

**DOI:** 10.3390/clinpract15010010

**Published:** 2025-01-02

**Authors:** Abir Fguirouche, Fatimazahra Ouahmani, Ikram Brahim, Raja Hazime, Nissrine Louhab, Najib Kissani, Mohamed Chraa, Brahim Admou

**Affiliations:** 1 Laboratory of immunology and HLA, Center of Clinical Research, Mohammed VI University Hospital, Marrakech 43150, Morocco; fatimazahra.oumhani@edu.uca.ac.ma (F.O.); ik.brahim@chumarrakech.ma (I.B.); ra.hazime@uca.ac.ma (R.H.); br.admou@uca.ac.ma (B.A.); 2 Biosciences Research Laboratory, Faculty of Medicine and Pharmacy, Cadi Ayyad University, Marrakech 40000, Morocco; 3 Department of Neurology, Mohammed VI University Hospital, Faculty of Medicine and Pharmacy, Cadi Ayyad University, Marrakech 40000, Morocco; ni.louhab@uca.ma (N.L.); na.kissani@uca.ma (N.K.); m.chraa@uca.ac.ma (M.C.)

**Keywords:** multiple sclerosis, HLA loci, predisposing, protective, frequency, Morocco

## Abstract

**Background:** Many factors contribute to the development and the progression of Multiple Sclerosis (MS), including Human Leukocyte Antigen (HLA) molecules. Some of them are considered as predisposing, like DRB1*15, DRB1*13, DRB1*03, DRB1*04, DQB1*06, DQB1*02, while HLA A2, HLA B44, DRB1*11, and DRB1*12 are rather considered as protective. Data about such associations in the Moroccan population remain unknown. The aim of this study was to determine the frequency of HLA class I (A and B) and II (DR and DQ) linked to Multiple Sclerosis (MS) in a healthy population from the South of Morocco. **Materials and Methods:** A cross-sectional study was carried out over the 2016–2023 period on 685 Moroccan healthy individuals, including 355 males and 330 females. Of the total sample tested, 685 underwent HLA class I typing, of which 305 also benefited from HLA class II typing. HLA class I typing was executed using the CDC (complement dependent cytotoxicity) technique (OneLambda™, Los Angeles CA, USA), and HLA class II typing was performed by either PCR-SSP (sequence-specific primer, OneLambda) or PCR-SSO (sequence-specific oligonucleotides) using the Luminex Xmap (Lifecodes, Immucor, Peachtree, Corners, GA, USA) system. **Results:** From different HLA molecules potentially predisposing to MS, our investigations showed that DRB1*03, DRB1*13, DRB1*15, DRB1*04, and DQB1*02 were observed in 19.2%, 15.8%, 13.31%, 12.7% and 31% respectively, while the frequency of those considered as protective, namely HLA-A2, HLA-B44, and HLA-DRB1*11 was 23.31%, 9.21% and 10.1% respectively. **Conclusions:** The findings of our study give evidence that among predisposing HLA class II molecules, DR allele groups were more prevalent, mostly DRB1*03, with also a high frequency of DQB1*06, while HLA-A2 marked the supposed protective specificities. These results need to be supported by complementary studies particularly in MS patients.

## 1. Introduction

Multiple Sclerosis (MS) is an inflammatory, chronic, and neurodegenerative disease affecting the Central Nervous System (CNS), due to dysregulations in immune response mechanisms [1]. MS occurs in young adults and is one of the main causes of non-traumatic handicap, leading to disabilities in approximately 2.8 million individuals worldwide with a stable prevalence over recent decades [2]. The World Health Organization (WHO) has estimated the prevalence of the disease in North African countries (NA) at 20 cases per 100.000 inhabitants [3].

MS is a multifactorial disease characterized by cellular immune responses, occurring in genetically predisposed individuals. The onset of the disease is often due to a complex interaction between genetic predisposition and environmental factors, requiring further investigation to fully understand its underlying mechanisms [3]. Many studies have highlighted the involvement of the human leukocyte antigen (HLA) system in the pathogenesis of MS since HLA molecules are responsible for presenting antigenic peptides to CD4+ and CD8+ T lymphocytes [4,5]. MS is related to both class I and class II alleles due to their crucial function within the immune system. It involves the presentation of peptides derived from extracellular proteins to immunocompetent cells [6].

The association of MS with HLA has been investigated in many studies, which showed that MS susceptibility is linked to the HLA-DRB1 gene, accounting for as much as 10.5% of the genetic variability associated with the susceptibility to MS [5]. It was first shown that HLA-A*02:01 is independently protective against MS, and later in 2018, when the Next Generation Sequencing (NGS) studies conducted in Caucasians showed that HLA-C*03:04 and HLA-B*40:01 alleles were also protective against MS, independently from the HLADRB1*15:01 allele. The latter accounts for 20% to 50% of the predisposition to the disease. Additional correlations with HLA-DR3, HLA-DR4 or HLA-DR13 have also been documented in the literature [3]. It is noteworthy that the HLA class I and II alleles vary between populations and even according to their ethnicity. This variation may affect directly the epidemiological aspects of MS [6].

Up to now, studies on the frequency of either predisposing or protecting HLA alleles associated with MS, especially in the Middle East and North Africa (MENA) regions, are scarce. The study of genetic polymorphisms within HLA loci in these populations could help identify susceptible populations, with a view to defining more effective therapeutic strategies tailored to specific immunogenic profiles and/or preventative measures.

The aim of this study was to determine the frequency of HLA class I and II loci potentially associated with MS in a healthy population from the south of Morocco.

## 2. Materials and Methods

### 2.1. Study Population Selection

We retrospectively enrolled a total of 685 healthy Moroccan individuals during a period of 7 years (2016–2023). All included individuals corresponded to donor candidates for organ, bone marrow, or HSC (hematopoietic stem cells). These individuals came from different regions of Southern Morocco, including Marrakech-Safi, Souss-Massa, Guelmim-Oued Noun, and Drâa-Tafilalet governorates to create a representative dataset of HLA diversity within the region since the studied population was characterized by the predominance of Berber/Amazigh populations in this region, alongside Arab and sub-Saharan African ancestries due to historical migrations and genetic admixture. Inclusion criteria were based on the absence of previous medical history or contraindications to transplantation and negative tests for major viral infections, including human immunodeficiency virus (HIV), hepatitis B virus (HBV), and hepatitis C virus (HCV). Patients who were on the recipient waiting list for organ, bone marrow or HSC transplant were excluded from this study. Among the included individuals, 685 underwent HLA class I typing, of which 305 also benefited from HLA class II typing.

Clinical data were collected using a datasheet indicating gender, age, origin, and full medical history of the population studied.

### 2.2. Sample Collection

Samples were collected from peripheral venous blood in two 9 mL lithium heparin tubes used for HLA class I typing, and two 5 mL tubes of Ethylenediaminetetraacetic Acid (EDTA) used for HLA class II typing. EDTA is an anticoagulant agent which guarantees the stabilization and preservation of various cellular subpopulations essential for HLA typing. All specimens were then immediately placed into appropriate transport containers and shipped to the laboratory under controlled conditions to maintain sample integrity. They were processed within 24h of collection, in order to retain the best quality DNA from the samples for future analyses.

### 2.3. Extraction of Genomic DNA

Genomic DNA was isolated from peripheral blood specimens (mononuclear cells) in accordance with the protocol established by the manufacturer, utilizing the QIAamp DNA Mini Kit (Qiagen, Hilden, Germany). This methodology encompassed cellular lysis, where mononuclear cells were treated with a chaotropic salt buffer designed to compromise cellular membranes and facilitate the liberation of DNA. A silica-based column was employed to process the lysate, allowing for the selective binding of DNA to the silica membrane, which enabled the removal of impurities such as salts, metabolites, and additional contaminants through a series of ethanol-based wash buffers, ultimately resulting in DNA of exceptional purity suitable for subsequent applications. The concentration and purity of the DNA were evaluated utilizing the NanoDrop™ 2000/2000c Spectrophotometer (Thermo Scientific™, Waltham, MA, USA). Quality control parameters (absorbance ratios at 260/280 nm and 260/230 nm) were documented to ascertain the DNA’s adequacy for HLA typing.

### 2.4. HLA Genotyping Analysis

HLA class I typing (HLA-A and HLA-B) was performed using the standardized complement-dependent cytotoxicity (CDC) method, employing the Terasaki HLA Tissue Typing Tray (One Lambda™, Los Angeles, CA, USA). This traditional, well-established methodology involves incubating lymphocytes derived from patients with specific anti-HLA sera, followed by the addition of complement. The occurrence of cellular apoptosis indicates a match between the serum antibody and the HLA antigen expressed on the lymphocyte. The assay results were interpreted microscopically to determine HLA-A and HLA-B specificities using the Lambda Monoclonal Typing AB Tray Worksheet provided by One Lambda™.

For HLA class II typing, high-resolution (allele-level) molecular methods were employed to meet the need for greater specificity and sensitivity. Specifically, PCR-SSP and PCR-SSO techniques were utilized. PCR-SSP was used to identify HLA-DRB1 and HLA-DQB1 alleles with allele-specific primers designed using Fusion Software 4.6.x (One Lambda™). This method involved amplification with primers specific to distinct HLA sequences, followed by electrophoretic analysis to identify alleles. PCR-SSO typing was performed using Luminex^®^ 200 technology (Immucor™), which hybridized fluorescently labeled oligonucleotide probes with amplified DNA. HLA alleles were assigned using the Xponent^®^ 3.1 analysis software (Immucor^®^, Peachtree, Corners, GA, USA), based on fluorescence intensity patterns.

### 2.5. Data Input and Statistical Analysis

Socio-demographic and clinical data of the study population were initially entered into an Excel database, then transferred and coded on the IBM SPSS statistic 23 platform to guarantee reliable statistical analysis and vigorous analysis of the data.

Frequencies of HLA class I and II specificities and allele groups were calculated, considering a double group allele for each individual, corresponding to either homozygous- or heterozygous-inherited HLA specificity. Potential MS linked HLA-A, -B, -DR and -DQ were identified.

### 2.6. Ethical Consideration

The samples examined were obtained through the standard practice of the HLA laboratory. The Electronic database of the laboratory served to retrieve the sociodemographic information under the supervision of the laboratory manager. Consequently, ethical authorization and informed consent were deemed superfluous for this specific case.

## 3. Results

### 3.1. Demographic and Social Attributes of the Population

The study population was composed of 66.8% adults and 33.2% children (<18 years), and the overall mean age was 27 ± 15 years. The 29–39 age group was predominant, accounting for 44.08% of cases (Table 1). There was a slight predominance of male gender, with a sex-ratio of 1.07.

Among the 685 cases studied, 610 had available data regarding their geographical origins. The region with the highest representation was Marrakech-Safi, accounting for 56.55%. Table 2 presents the geographical origins of the individuals included in the study.

### 3.2. Frequencies of HLA Class I (A, B) and Class II (DR, DQ) Allele Categories

As exhibited in Table 3, the distribution of HLA class I and II among the population shows that within each HLA locus, A2, B51, DRB1*03, and DQB1*02 were the most prevalent allele groups, at about 23%, 10%, 19%, and 31%, respectively.

#### 3.2.1. HLA Class I Typing Results

Out of the 684 individuals who underwent HLA-A class I typing, the distribution of allele groups showed that HLA-A2 was the most frequent, at 23.3%, followed by A1 (11.6%), A3 (8.9%), A23 (7%), and A24 (7), while HLA-A68 and HLA-A30 frequencies were less frequent, at 7.8% and 6.5%, respectively.

Among HLA-B locus allele groups, B51 was the most prevalent, displaying a value of 9.9%, followed by B44 (9.1%), B8 (6.7%), B49 (6.4%), and then B45 (6%) (Table 3).

#### 3.2.2. HLA-A and HLA-B Frequencies According to Gender Haut du Formulaire

According to gender, we noticed a predominance of the A2 allele group in the female population at 23.6%, followed by A1 (12.3%), A3 (9.4%), A68 (8%), then A24 (7.6%), and A23 (7%). In the male population, we observed a predominance of HLA-A2 allele groups with a frequency of 23.1%, followed by A1 (11%), A23 (8.8%), A3 (8.4%), then A24 (8.3%), and A30 (7.6%).

Regarding the HLA-B locus results, the most common allele groups among the female group was B51 with 10.94%, followed by B49 (8.21%), B44 (8%), B8 (7.29%), and B35 (6.38%). In males, the prevalence of HLA-B44 was estimated at 10.28%, followed by B51 (9%), B7(6.7%), and B45 (6.2%) (Table 4).

HLA-B49 and HLA-B57 were the only allele groups to show a significant association according to gender in favor of females (*p* =0.023 and *p* =0.027, respectively).

### 3.3. HLA Class II Typing Analysis

Among the 305 cases that benefited from HLA-DRB1 typing, a total of 610 alleles were analyzed. The most widespread allele groups seen were HLA-DRB1*03 (19.2%), followed by DRB1*13 (15.8%), DRB1*07 (14.9%) then, DRB1*15 (13.3%) (Table 3). Concerning the HLA-DQB1 locus analysis, the most frequent allele groups were HLA-DQB1*02 (31.4%), DQB1*06 (24.3%), then DQB1*03(24%) and, DQB1*05 (11.8%) as described in Table 3.

#### HLA-DR and HLA-DQ Frequencies According to Gender

In the female population, HLA-DRB1*03 was predominant, with a frequency of 20.18%, followed by DRB1*07 (16.8%), DRB1*13 (15.6%), then DRB1*04 (12.3%). In the male population, the allele HLA-DRB1*03 emerged as the predominant variant, accounting for 18.15%, succeeded by both DRB1*13 and DRB1*15, each comprising 15.92%, followed subsequently by DRB1*04, which represented 13.06%.

HLA-DQ typing within the female population showed that HLA-DQB1*02 emerged as the most frequent allele group (31.4%), followed by DQB1*03 (24.1%) then DQB1*06 (20.7%). In males, HLA-DQB1*02 was present in 28% of the studied group, followed by DQB1*06 (28%), and DQB1*03 (24.1%) (Table 5).

The frequencies of HLA-DRB1*07 and HLA-DQB1*02 were highly significant in females, with *p*-values of 0.042 and 0.018, respectively.

## 4. Frequency of HLA Class I and II Loci Linked to Multiple Sclerosis

### 4.1. Frequency of HLA Class I Loci Known to Be Linked to Multiple Sclerosis

In our study, the HLA predisposing class I allele group frequencies were A3 (8.9%), B7 (4.56%), and then B27 (3.7%), while the most predominant protective HLA frequencies were A2 (23.3%), B52 (9.1%), and B44 (1.2%).

There was no statistically significant difference observed concerning HLA specificities associated with MS.

### 4.2. Frequency of HLA Class II Loci Known to Be Linked to Multiple Sclerosis

HLA class II molecules potentially predisposing to MS allele group frequencies were DRB1*03, at 19.2%, DRB1*15 (13.3%), then DRB1*13 (15.8%) and DRB1*04 (12.7%). The frequency of protective allele groups was 7.4% for DRB1*01 and 10.1% for DRB1*11.

Concerning predisposing HLA-DQ loci, DQB1*02 was significantly more frequent in females with 31.4% (*p* =0.018), while the frequency of DQB1*06 was also high, at 24.3%.

## 5. Discussion

Our study was conducted on 685 healthy individuals who were originated from the south of Morocco, characterized by a dominance of Berber/Amazigh, alongside Arab and sub-Saharan African ancestries, and contributing to great ethnic diversity in this geographical region. This diversity is due to historical migratory flows and the strategic geographic position of Morocco, which have probably contributed to the polymorphic genetic background.

The study evaluated the frequency of the main HLA class I (A, B) and class II (DQ, DR), possibly considered as susceptibility or protective factors against MS in a healthy population.

According to the literature, HLA class I polymorphism seems to be less studied compared to HLA class II in MS since many class I alleles appear to be secondary to class II associations [6,7]. Studies from the 70s have reported the earliest relationship between MS and HLA, especially HLA class I alleles. Using serological based techniques, it was shown that A3, B7 and B27 are predisposing alleles with high risk of MS [8,9,10] while A2, B52, and B44 are associated with reduced susceptibility in many populations [11,12,13].

The results of the current study highlighted the emergence of HLA-A2 (23.31%), HLA-A1 (11.62%), B51 (9.94%), and B44 (9.21%) as the most frequent loci within the population. These observations are in accordance with frequencies previously described in other Moroccan studies [14,15].

In other countries from the MENA region, like Iran and Iraq, HLA-A2 and B44 are also more prevalent, with high frequencies among healthy controls [16,17], and are likely protective against MS. Additionally, Cohorts from Norway [18] and Italy [19] have also demonstrated a protective role for A2. Despite the consistency in the results confirmed by numerous studies, the frequencies of A2, A3, A10, and A11 reportedly differ between populations, and even within the same population. For instance, Ghabaae et al., had attributed a protective role against MS to HLA-A11, while Lotfi et al. reported an increased risk of MS within another Iranian population [10,20]. Furthermore, HLA-B7, B27 and A3, observed with lower frequencies in our study, are generally considered as predisposing to MS. However, the analysis of allele transmission in Canadians assumed that any effect attributable to A3 is secondary to linkage disequilibrium (LD) with class II alleles. Similarly, HLA-A3 is in sturdy LD with HLA-B7. Therefore, any association observed for HLA-B7 is due to LD with the extended class II alleles [21].

Pertaining to the HLA class II results, the high frequencies of HLA-DRB1*03 (19.2%), DRB1*13 (15.8%), DRB1*07 (14.86%), and DRB1*04 (12.7%) found in our study are also described as susceptibility factors of MS in several groups within different ethnic backgrounds, notably Arabs [5,6]. These findings align with other studies driven in healthy populations, mainly from Tunisia, Europe, and Asia [6,22]. Among MS-related HLA-DR alleles that have been reported in the literature, DRB1*15 represents the highest MS risk factor in the HLA region across several ethnicities. This allele was present in 13.3% of our population, the same as in Italy (11%), Spain (29%) [23], France (26%), Turkey (21%), and in Northern Europe populations [23,24]. Moreover, molecular analysis studies have proven shared amino acids between DRB1*15:01, DRB1*03:01, and DRB1*04:03 alleles, conferring MS susceptibility by playing an important role in autoantigen presentation to T lymphocytes [3,25]. The DRB1*03:01 allele has been detected in MS patients from Morocco and Sardinia [3,26]. In Morocco, DRB1*13 was seen in 12% of healthy controls and 7.9% of diseased populations [3], whereas it is rarely observed at frequencies greater than 3% worldwide [6]. Regarding HLA-DQ specificities, our study revealed high frequencies of DQB1*02 (31.4%) and DQB1*06 (24.3%), which align with data reported by Brick et al. [14] and Izaabel et al. [27]. However, the DQB1*06 allele was more predominant in MS patients than in controls in European and Caucasian populations, unlike Sardinians and Italians [28,29]. The demographic composition of the MENA region demonstrated a comparable distribution of the HLA-DRB1 alleles to adjacent ethnic cohorts, revealing heightened genetic similarities with Western populations and individuals of Caucasian descent, in contrast to those originating from Eastern Asia, African-American groups, or Latin-American populations [30].

To sum up, at the limits of our knowledge, our study is the first to highlight the distribution of main HLA allele groups in relation to MS in a healthy population from a large geographical part of Morocco. However, despite their originality, these findings are preliminary and need to be further supported by investigations, particularly in MS patients with a stronger effect size. This could facilitate the development of personalized medical approaches, enabling targeted interventions that take into account individual genetic and environmental aetiologies. Our study can potentially lead to improvements in diagnostic and therapeutic strategies. Furthermore, it could enhance patient outcomes by adapting treatments to the unique genetic and environmental landscape of each individual, ultimately fostering more effective management of MS.

## 6. Conclusions

Given the importance of knowing the factors potentially linked to MS, including the immunogenic background of a healthy Moroccan population, our study highlighted high frequencies of HLA-A2, DRB1*03, DRB1*15, DRB1*11, DQB1*02, and DRB1*06. Particular predisposing HLA allele groups were notably prevalent in our population, like DRB1*03, DRB1*13, DRB1*15, DRB1*04, and DQB1*02, whereas those considered as protective, namely HLA-A2, HLA-B44, and HLA-DRB1*11, were also predominant inside a heterogeneous HLA landscape. These data could help build a local HLA database useful for better management of MS and other HLA-related diseases.

Understanding these genetic variations is essential for developed and individualized therapeutic approaches, which would enhance preventive strategies customized for particular demographic groups. It is important to consider genetic diversity in clinical trials, as this can lead to more effective interventions and a better understanding of the pathomechanisms of MS.

## Figures and Tables

**Table 1:** Demographic and social attributes of the population.

Age Group (Years)	Number of Cases	Frequency %
**≤15**	121	17.66
**16–28**	110	16.05
**29–39**	302	44.08
**≥40**	152	22.19

**Table 2:** Geographic origins of the studied population.

Geographic Area	Number of Cases	Frequency %
Marrakech-Safi	345	56.55
Souss-Massa	98	16.06
Daraa-Tafilalt	53	8.68
Dakhla-Oued Eddahab	52	8.52
Guelmim-Oued Noun	37	6.06
Laayoune Sakia Hamra	25	4.09

**Table 3:** Frequency distribution of HLA Class I (A, B) and Class II (DR, DQ) within our series.

Class I				Class II			
HLA-A	Frq (%)	HLA-B	Frq (%)	HLA-DR	Frq (%)	HLA-DQ	Frq (%)
**A2**	23.3	**B51**	9.9	DRB1*03	19.2	DQB1*02	31.4
**A1**	11.6	**B44**	9.1	DRB1*13	15.8	DQB1*06	24.3
**A3**	8.9	**B8**	6.7	DRB1*07	14.8	DQB1*03	24
**A23**	7	**B49**	6.4	DRB1*15	13.4	DQB1*05	11.8
**A24**	7	**B45**	6	DRB1*04	12.8	DQB1*04	5
**A33**	3.7	**B35**	6.4	DRB1*11	10.6	DQB1*07	0
**A11**	3.3	**B18**	4.2	DRB1*01	7.1		
**A32**	3.7	**B58**	4.2	DRB1*08	2		
**A29**	3.1	**B27**	3.7	DRB1*09	1.6		
**A26**	2.6	**B7**	4.56	DRB1*10	0.5		
**A34**	2.3	**B38**	3.1	DRB1*14	0.5		
**A80**	1.7	**B53**	2.6	DRB1*12	0		
**A31**	1.1	**B41**	2.4	DRB1*16	0		
**A66**	1.4	**B57**	2.3				
**A68**	7.8	**B42**	2.1				
**A74**	0.8	**B63**	2.1				
**A36**	0.2	**B39**	2				
**A69**	0.2	**B72**	1.7				
**A25**	0.1	**B52**	1.2				
**A68**	7.8	**B13**	1				
**A30**	6.5	**B2**	0.1				
		**B37**	0.7				
		**B78**	0.7				
		**B47**	0.4				
		**B55**	0.2				
		**B56**	0.2				
		**B62**	0.2				
		**B64**	0.1				
		**B65**	0.1				
		**B71**	0.1				
		**B73**	0.1				

**Table 4:** Distribution of HLA class I allele groups according to gender.

Female (*n* =329, Allele *n* =658)				Male (*n* =355, Allele *n* =710)			
HLA-A	Allele Frq %	HLA-B	Allele Frq %	HLA-A	Allele Frq %	HLA-B	Allele Frq %
A1	**12.31**	**B2**	-	**A1**	**11**	**B2**	0.14
A2	**23.6**	**B7**	4.56	**A2**	**23.1**	**B7**	**6.76**
A3	**9.42**	**B8**	**7.29**	**A3**	**8.45**	**B8**	5.92
A11	3.65	**B13**	1.22	**A11**	3.66	**B13**	0.85
A23	7	**B18**	4.26	**A23**	8.87	**B18**	4.23
A24	7.6	**B35**	6.38	**A24**	8.31	**B35**	5.07
A25	0.15	**B37**	0.76	**A25**	0.14	**B37**	0.56
A26	2.43	**B38**	2.28	**A26**	2.82	**B38**	3.94
A29	2.58	**B41**	2.13	**A29**	3.66	**B41**	2.68
A30	5.32	**B42**	1.82	**A30**	7.61	**B42**	2.39
A31	0.91	**B44**	**8.05**	**A31**	1.55	**B44**	**10.28**
A32	4.26	**B45**	5.78	**A32**	3.1	**B45**	6.2
A33	4.1	**B47**	0.15	**A33**	3.94	**B47**	0.7
A34	2.28	**B49**	**8.21**	**A34**	2,25	**B49**	4.79
A36	0.46	**B50**	5.78	**A36**	-	**B50**	4.65
A66	1.06	**B53**	2.58	**A66**	0.85	**B53**	2.68
A68	8.05	**B52**	0.76	**A68**	7.61	**B52**	1.69
A69	0.46	**B55**	0.3	**A69**	-	**B55**	0.14
A74	0.76	**B57**	3.34	**A74**	0.85	**B57**	1.27
A80	1.98	**B58**	3.19	**A80**	1	**B58**	5.07
		**B63**	2.74			**B63**	1.55
		**B56**	0.15			**B56**	0.28
		**B62**	0.15			**B62**	0.28
		**B64**	0.3			**B64**	-
		**B65**	-			**B65**	0.14
		**B73**	0.15			**B73**	-
		**B71**	-			**B71**	0.14
		**B72**	1.52			**B72**	1.83
		**B78**	0.46			**B78**	0.85

**Table 5:** Distribution of HLA class II allele groups according to gender.

Female (*n* =155, Allele *n* =310)				Male (*n* =150, Allele *n* =300)			
HLA-DR	Allele Frq %	HLA-DQ	Allele Frq %	HLA-DR	Allele Frq %	HLA-DQ	Allele Frq %
DRB1*01	8.43	DQB1*02	31.4	DRB1*01	8.43	DQB1*02	28
DRB1*03	20.18	DQB1*03	24.1	DRB1*03	20.18	DQB1*03	24.1
DRB1*04	12.35	DQB1*04	4.7	DRB1*04	12.35	DQB1*04	5.72
DRB1*07	16.87	DQB1*05	12.25	DRB1*07	16.87	DQB1*05	10.25
DRB1*08	1.9	DQB1*06	20.78	DRB1*08	2.11	DQB1*06	28
DRB1*09	1.2	DQB1*07	-	DRB1*09	1.2	DQB1*07	-
DRB1*10	0			DRB1*10	0.5		
DRB1*11	10			DRB1*11	10.84		
DRB1*13	15.66			DRB1*13	15.66		
DRB1*14	0.6			DRB1*14	0.6		
DRB1*15	10.84			DRB1*15	10.84		

## Data Availability

The data in this study are not publicly available due to privacy restrictions. Researchers interested in accessing the data for replication or verification may request it through the institutional review board, subject to approval and privacy compliance. For data access inquiries, please contact Pr. Brahim ADMOU at the Clinical Research Center, Mohammed VI University Hospital Center (email: br.admou@uca.ac.ma).

## References

[1] Hauser S.L., Goodin D.S., Longo D.L., Fauci A.D., Kasper D.L., Hauser S.L., Jameson J.L., Loscalzo J. (2012). Multiple sclerosis and other demyelinating diseases. Harrison’s Principle of Internal Medicine.

[2] Batran R.A., Kamel M., Bahr A., Waheb J., Khalil A., Elsokary M. (2024). Multiple Sclerosis: Economic Burden, Therapeutic Advances, and Future Forecasts in the Middle East and North Africa Region. Expert Rev. Pharmacoecon. Outcomes Res..

[3] Ouadghiri S., Toussi K.E.A., Brick C., Benhaddou E.A., Benseffaj N., Benomar A., El Yahyaoui M., Essakalli M. (2013). Genetic factors and multiple sclerosis in the Moroccan population: A role for HLA class II. Pathol. Biol..

[4] Grau-López L., Raïch D., Ramo-Tello C., Naranjo-Gómez M., Dàvalos A., Pujol-Borrell R., Borràs F.E., Martínez-Cáceres E. (2009). Myelin peptides in multiple sclerosis. Autoimmun. Rev..

[5] Maghbooli Z., Sahraian M.A., Naser Moghadasi A. (2020). Multiple sclerosis and human leukocyte antigen genotypes: Focus on the Middle East and North Africa region. Mult. Scler. J. Exp. Transl. Clin..

[6] Hollenbach J.A., Oksenberg J.R. (2015). The immunogenetics of multiple sclerosis: A comprehensive review. J. Autoimmun..

[7] Shiina T., Hosomichi K., Inoko H., Kulski J.K. (2009). The HLA genomic loci map: Expression, interaction, diversity and disease. J. Hum. Genet..

[8] Jersild C., Hansen G., Svejgaard A., Fog T., Thomsen M. (1973). Histocompatibility determinants in multiple sclerosis, with special reference to clinical course. Lancet.

[9] Jersild C., Svejgaard A., Fog T. (1972). HL-A antigens and multiple sclerosis. Lancet.

[10] Ghabaee M., Bayati A., Saroukolaei S.A., Sahraian M.A., Sanaati M.H., Karimi P., Houshmand M., Sadeghian H., Chelavi L.H. (2009). Analysis of HLA DR2&DQ6 (DRB1*1501, DQA1*0102, DQB1*0602) haplotypes in Iranian patients with multiple sclerosis. Cell Mol. Neurobiol..

[11] Link J., Kockum I., Lorentzen R., Lie B.A., Celius E.G., Westerlind H., Schaffer M., Alfredsson L., Olsson T., Brynedal B. (2012). Importance of human leukocyte antigen (HLA) class I and II alleles on the risk of multiple sclerosis. PLoS ONE.

[12] Bergamaschi L., Ban M., Barizzone N., Leone M., Ferrante D., Fasano M.E., Guerini F.R., Corrado L., Naldi P., Dametto E. (2011). Association of HLA class I markers with multiple sclerosis in the Italian and UK population: Evidence of two independent protective effects. J. Med. Genet..

[13] Benedek G., Paperna T., Avidan N., Lejbkowicz I., Oksenberg J.R., Wang J., Brautbar C., Israel S., Miller A. (2010). Opposing effects of the HLA-DRB1*0301-DQB1*0201 haplotype on the risk for multiple sclerosis in diverse Arab populations in Israel. Genes Immun..

[14] Brick C., Bennani N., Atouf O. (2006). HLA-A,-B,-DR and-DQ allele and haplotype frequencies in the Moroccan population: A general population study. Trans. Clin. Biol..

[15] Piancatelli D., Canossi A., Aureli A., Oumhani K., Del Beato T., El Aouad R., Adorno D. (2004). HLA-A, -B, -Cw and -DRB1 allele frequencies in a Metalsa population from Nador, Morocco. Hum. Immunol..

[16] Amirzargar A.A., Tabasi A., Khosravi F., Kheradvar A., Rezaei N., Naroueynejad M., Ansaripour B., Moradi B., Nikbin B. (2005). Optic neuritis, multiple sclerosis and human leukocyte antigen: Results of a 4-year follow-up study. Eur. J. Neurol..

[17] Saleem M.A., Mukhelif H.F., Moussawi K.M., Al-Khafaji J.T. (2007). Human leukocyte antigen typing in Iraqi multiple sclerosis patients. Neurosciences.

[18] Harbo H., Lie B., Sawcer S., Celius E., Dai K., Oturai A., Hillert J., Lorentzen Å.R., Laaksonen M., Myhr K. (2004). Genes in the HLA class I region may contribute to the HLA class II-associated genetic susceptibility to multiple sclerosis. Tissue Antigens.

[19] Bergamaschi L., A Leone M., E Fasano M., Guerini F.R., Ferrante D., Bolognesi E., Barizzone N., Corrado L., Naldi P., Agliardi C. (2009). HLA-class I markers and multiple sclerosis susceptibility in the Italian population. Genes Immun..

[20] Lotfi J., Nikbin B., Derakhshan I., Aghai Z., Ala F. (1978). Histocompatibility antigens (HLA) in multiple sclerosisin Iran. J. Neurol. Neurosurg. Psychiatry.

[21] Chao M.J., Barnardo M.C., Lui G.Z., Lincoln M.R., Ramagopalan S.V., Herrera B.M., Dyment D.A., Sadovnick A.D., Ebers G.C. (2007). Transmission of class I/II multi-locus MHC haplotypes and multiple sclerosis susceptibility: Accounting for linkage disequilibrium. Hum. Mol. Genet..

[22] Messadi A., Najiba F.M., Ouerhani S., Zaweli J., Louatti I., Layouni S., Nciri B., Bouaicha G., Kouki W., Yedeas M. (2010). HLA class II alleles and multiple sclerosis in Tunisian patients. Clin. Neurol. Neurosurg..

[23] Fernández O., R-Antigüedad A., Pinto-Medel M.J., Mendibe M.M., Acosta N., Oliver B., Guerrero M., Papais-Alvarenga M., Fernández-Sánchez V., Leyva L. (2009). HLA class II alleles in patients with multiple sclerosis in the Biscay province (Basque Country, Spain). J. Neurol..

[24] Silva A.M., Pereira C., Bettencourt A., Carvalho C., Couto A.R., Leite M.I., Marta M., Freijo M., Costa P.P., Mendonça D. (2007). The role of HLA-DRB1 alleles on susceptibility and outcome of a Portuguese multiple sclerosis population. J. Neurol. Sci..

[25] Haegert D.G., Francis G.S. (1992). Contribution of a single DQ_ chain residue to multiplesclerosis in French Canadians. Hum. Immunol..

[26] Marrosu M.G., Murru M.R., Costa G., Cucca F., Sotgiu S., Rosati G., Muntoni F. (1997). Multiple sclerosis in Sardinia is associated and in linkage disequilibrium with HLA-DR3 and -DR4 alleles. Am. J. Hum. Genet..

[27] Izaabel H., Garchon H., Caillat-Zucman S., Beaurain G., Akhayat O., Bach J., Sanchez-Mazas A. (1998). HLA class II DNA polymorphism in a Moroccan population from the Souss, Agadir area. Tissue Antigens.

[28] Marrosu M.G., Murru M.R., Costa G., Murru R., Muntoni F., Cucca F. (1998). DRB1–DQA1–DQB1 loci and multiple sclerosis predisposition in the Sardinian population. Hum. Mol. Genet..

[29] Zivadinov R., Uxa L., Zacchi T., Nasuelli D., Ukmar M., Furlan C., Pozzi-Mucelli R., Tommasi M.A., Locatelli L., Ulivi S. (2003). HLA genotypes and disease severity assessed by magnetic resonance imaging findings in patients with multiple sclerosis. J. Neurol..

[30] Mohajer B., Abbasi N., Pishgar F., Abdolalizadeh A., Ebrahimi H., Razaviyoun T., Mohebbi F., Eskandarieh S., Sahraian M.A. (2018). HLA-DRB1 polymorphism and susceptibility to multiple sclerosis in the Middle East North Africa region: A systematic review and meta-analysis. J. Neuroimmunol..

